# Integrated smoking cessation and mood management following acute coronary syndrome: Protocol for the post-acute cardiac event smoking (PACES) trial

**DOI:** 10.1186/s13722-023-00388-9

**Published:** 2023-05-12

**Authors:** Melissa Adkins-Hempel, Sandra J. Japuntich, Michelle Chrastek, Shira Dunsiger, Christopher E. Breault, Woubeshet Ayenew, Susan A. Everson-Rose, Prabhjot S. Nijjar, Beth C. Bock, Wen-Chih Wu, Michael D. Miedema, Brett M. Carlson, Andrew M. Busch

**Affiliations:** 1 Behavioral Health Equity Research Group, Hennepin Healthcare Research Institute, 701 Park Ave. S9.104, Minneapolis, MN 55415 USA; 2 Division of Clinical Pharmacology, Department of Medicine, Hennepin Healthcare, 900 S. 8th St., G5, Minneapolis, MN 55415 USA; 3 Division of General Internal Medicine, Department of Medicine, University of Minnesota, 401 East River Parkway, Suite 131, Minneapolis, MN 55455 USA; 4 Department of Behavioral and Social Sciences, Center for Health Promotion and Health Equity, School of Public Health, Brown University, 121 South Main St., Providence, RI 02903 USA; 5 Department of Epidemiology, School of Public Health, Brown University, 121 South Main St., Providence, RI 02903 USA; 6 Center for Behavioral and Preventative Medicine, Lifespan, 1 Hoppin St., Suite 309, Providence, RI 02903 USA; 7 Division of Cardiology, Department of Medicine, Hennepin Healthcare, 900 South 8th St., O5, Minneapolis, MN 55415 USA; 8 Cardiovascular Division, Department of Medicine, University of Minnesota Medical School, 420 Delaware Street SE, MMC 508, Minneapolis, MN 55455 USA; 9 Program in Health Disparities Research, University of Minnesota, 717 Delaware St. SE, Suite 166, Minneapolis, MN 55414 USA; 10 Department of Psychiatry and Human Behavior, Alpert School of Medicine, Brown University, 700 Butler Drive, Providence, RI 02906 USA; 11 Center of Innovation in Long Term Services and Support, Providence VA Medical Center, 830 Chalkstone Ave., Providence, RI 02908 USA; 12 Cardiovascular Rehab Center, Lifespan, 208 Collyer St., Providence, RI 02904 USA; 13 Minneapolis Heart Institute and Minneapolis Heart Institute Foundation, 920 East 28th St., Suite 480, Minneapolis, MN 55407 USA; 14 North Memorial Health Heart and Vascular Center, 3300 Oakdale Ave. N., Suite 200, Robbinsdale, MN 55422 USA

**Keywords:** Smoking, Depression, Behavioral activation, Secondary prevention, Acute coronary syndrome, Cardiovascular disease

## Abstract

**Background:**

Approximately 400,000 people who smoke cigarettes survive Acute Coronary Syndrome (ACS; unstable angina, ST and non-ST elevation myocardial infarction) each year in the US. Continued smoking following ACS is an independent predictor of mortality. Depressed mood post-ACS is also predictive of mortality, and smokers with depressed mood are less likely to abstain from smoking following an ACS. A single, integrated treatment targeting depressed mood and smoking could be effective in reducing post-ACS mortality.

**Method/design:**

The overall aim of the current study is to conduct a fully powered efficacy trial enrolling 324 smokers with ACS and randomizing them to 12 weeks of an integrated smoking cessation and mood management treatment [Behavioral Activation Treatment for Cardiac Smokers (BAT-CS)] or control (smoking cessation and general health education). Both groups will be offered 8 weeks of the nicotine patch if medically cleared. Counseling in both arms will be provided by tobacco treatment specialists. Follow-up assessments will be conducted at end-of-treatment (12-weeks) and 6, 9, and 12 months after hospital discharge. We will track major adverse cardiac events and all-cause mortality for 36 months post-discharge. Primary outcomes are depressed mood and biochemically validated 7-day point prevalence abstinence from smoking over 12 months.

**Discussion:**

Results of this study will inform smoking cessation treatments post-ACS and provide unique data on the impact of depressed mood on success of post-ACS health behavior change attempts.

***Trial registration*:**

ClinicalTrials.gov, NCT03413423. Registered 29 January 2018. https://beta.clinicaltrials.gov/study/NCT03413423.

**Supplementary Information:**

The online version contains supplementary material available at 10.1186/s13722-023-00388-9.

## Background

1.2 million individuals survive acute coronary syndrome (ACS; unstable angina, ST and non-ST elevation myocardial infarction) in the US each year [1]. Cigarette smoking is a major risk factor for ACS and ≈ 400,000 survivors per year were smoking prior to ACS [2, 3]. ACS is a “teachable moment,” in that patients are more receptive to smoking cessation messages, and it provides an opportunity to practice abstinence during hospital stays [4]. Smoking cessation in ACS patients reduces mortality by 36–46% [5, 6].

Depressed mood is a major concern in ACS patients. Clinical depression and sub-clinical depression symptoms are much more common in ACS patients than the general population [7, 8]. Symptoms of depression, even when mild, independently predict post-ACS morbidity and mortality [9, 10], and the American Heart Association now recognizes depression as a major risk factor for poor prognosis following ACS [8].

Smokers are more likely to be depressed at the time of ACS and to experience onset of depression post-ACS than people without a smoking history [11–13]. Smokers with depressed mood are less likely to abstain from smoking following an ACS hospitalization [14]. Further, smoking status partially mediates the relationship between depression and subsequent mortality [15–18], suggesting that depressed mood contributes to mortality both directly *and* through interference with smoking cessation. Although there are counseling treatments that address smoking and depression in isolation in ACS patients, there is no integrated treatment that addresses both simultaneously. This is likely one reason why most ACS patients relapse to smoking within 1 year after ACS hospitalization [19, 20] and why treatments for depression post-ACS have resulted in less than expected improvement in morbidity and mortality rates [21]. A single, integrated treatment that targets both depressed mood and smoking could be highly effective in reducing post-ACS morbidity and mortality.

Behavioral Activation (BA) is a counseling intervention with clearly established efficacy for the treatment and prevention of depression in both psychiatric and medical populations [22–25]. Core BA techniques have been successfully applied as components of larger collaborative care interventions in multiple studies of post-ACS depression treatment [26–29]. BA is easier to train than other empirically-supported counseling treatments and can be delivered with fidelity by bachelor’s level practitioners [30–35] or even family members or peers [35]. BA has also recently shown promise for enhancing smoking cessation outcomes in people with elevated depression symptoms [36, 37].

Our team used a series of semi-structured interviews and test cases to systematically develop an intervention integrating gold standard smoking cessation counseling with existing BA based mood management techniques for post-ACS smokers; Behavioral Activation Treatment for Cardiac Smokers (BAT-CS) [38]. The team then conducted a pilot RCT of BAT-CS (n = 64), which demonstrated that the design proposed below is logistically feasible and highly acceptable, and that BAT-CS shows promise for efficacy on smoking cessation and mood outcomes at 6-month follow-up [38]. It is now necessary to establish the efficacy of BAT-CS in a rigorous, fully powered RCT with longer-term follow up.

A fully powered efficacy trial is underway which will enroll 324 daily smokers with ACS. Patients are randomized to 12 weeks of BAT-CS or a control group where counselors provide the same smoking cessation content plus health education content as a contact control for time spent on BA techniques. Both groups are offered the nicotine patch if medically safe. Follow-up assessments are conducted at end-of-treatment (12-weeks) and 6, 9, and 12 months after hospital discharge. We are tracking the occurrence of major adverse cardiac events and all-cause mortality for 36 months post discharge.

The primary aims and hypotheses of this study are:

### Aim I

Test the efficacy of BAT-CS versus control on depressed mood and smoking cessation. *Hypothesis 1a*: Patients treated with BAT-CS will demonstrate significantly greater improvements in mood and significantly higher rates of biochemically verified 7-day point prevalence abstinence over 12 months than control patients. This trial is powered for hypothesis 1a analyses. *Hypothesis 1b*: Patients treated with BAT-CS will also have higher rates of 30-day point prevalence and continuous abstinence since discharge and longer time to smoking lapse and relapse than control patients.

### Aim II

Test the efficacy of BAT-CS versus control on cardiac health. *Hypothesis 2a*: Patients treated with BAT-CS will have greater functional capacity and lower rates of major adverse cardiac events (i.e., hospitalization for unstable angina, urgent coronary revascularization, myocardial infarction) and all-cause mortality.

### Aim III

**Fig. 1:**
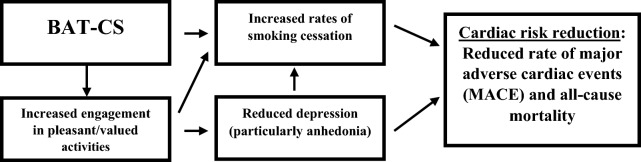
Conceptual model

Explore hypothesized mediators of treatment effects (see Fig. 1). *Hypothesis 3a*: Between group differences in depressed mood will be mediated by changes in BA theory-based mechanisms (i.e., greater engagement in pleasant and personally valued activities). *Hypothesis 3b*: Between group differences in smoking cessation will be mediated by both changes in depressed mood and BA theory-based mechanisms.

### Aim IV

Explore the temporal interaction of smoking and multiple aspects of mood over 1-year post-ACS.

## Methods

### Study design overview

**Fig. 2:**
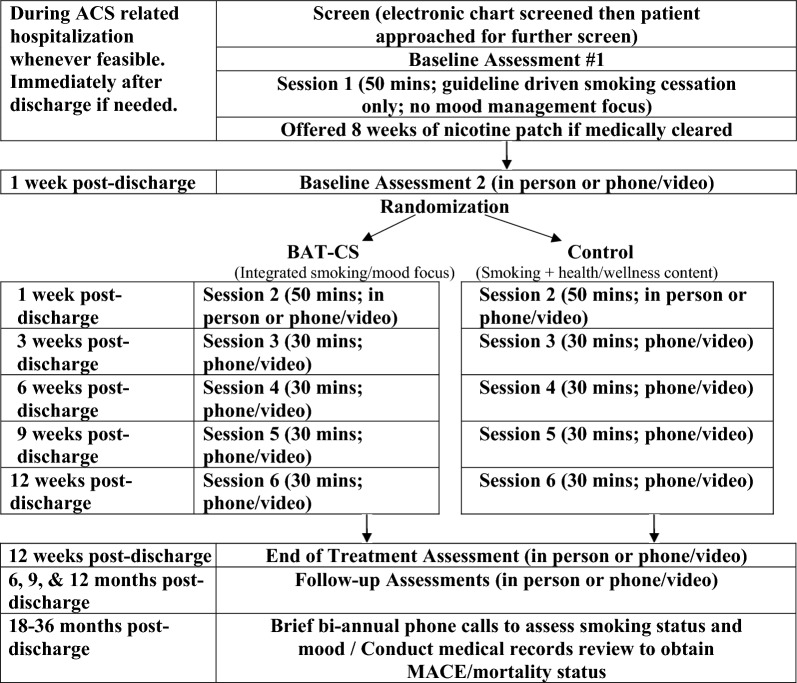
Design overview

324 ACS patients who smoke will be recruited and randomly assigned to 12 weeks of either Behavioral Activation Treatment for Cardiac Smokers (BAT-CS; smoking cessation and BA based mood management counseling) or a control condition (smoking cessation and health education) that fully controls for BA contact time (see Fig. 2). All participants will be offered 8 weeks of the nicotine patch if medically appropriate. All counseling in both conditions will be provided by tobacco treatment specialists over 12 weeks post-discharge. Comprehensive assessments completed by blind to condition assessors will occur at end-of-treatment (12 weeks after discharge) and 6, 9, and 12 months after discharge. Major Adverse Cardiac Events (MACE; i.e., hospitalization for unstable angina, urgent coronary revascularization, myocardial infarction) and all-cause mortality will be tracked through the end of the study for up to 36 months (see Table 1 and Fig. 2). Recruitment began on 1/22/2018. This protocol is presented consistent with SPIRIT guidelines (see additional file 1).

**Table 1:** Assessments by time point

	Screen	Baseline 1	Baseline 2	Each session	12 weeks/end-of-treatment	6, 9, 12 months	18, 24, 30, and 36 months
*Screening fully*							
Medical diagnosis, current smoking, and other inclusion/exclusion criteria	X						
Cognitive functioning (MMSE [43])	X						
*Participant characteristics*							
Demographics (age, race, etc.)		X					
Medical and smoking history		X					
Nicotine dependence (FTND [50])		X					
Motivation to quit (contemplation ladder [51])		X	X				
MN withdrawal scale [52]			X				
*Primary outcomes*							
Current smoking/smoking since last contact		X	X	X	X	X	X
Use of other tobacco products		X	X	X	X	X	X
Saliva sample for cotinine testing			X		X	X	
Exhaled breath sample for CO testing			X		X	X	
Depressive symptoms (PHQ-9 [53])		X	X		X	X	X
Depressive symptoms (CESD-10 [66])		X	X		X	X	
*Secondary outcomes*							
Positive and negative affect (PANAS [57])		X	X		X	X	X
Reactive anhedonia (SHAPS [68, 69])		X	X		X	X	
Perceived stress scale (PSS-4 [70])		X			X	X	
Health related quality of life (SF-12 [71])		X			X	X	
Exercise tolerance (DASI [72])		X			X	X	
MACE/mortality					X	X	X
Adverse event monitoring			X	X	X	X	X
*Hypothesized BA mediators*							
Pleasant/valued activities (BADS [76], PES [79, 80])		X			X	X	
*Acceptability*							
Treatment acceptability (CSQ-8 [81])					X		
*Potential confounders*							
Medication adherence questions [88, 89]		X			X	X	
Diet/exercise questions [92]		X			X	X	
Counseling sessions attended				X			
Cardiac rehabilitation attendance					X	X	
Outside of study depression treatment					X	X	
Outside of study smoking cessation treatment					X	X	

### Study setting

Recruitment will be conducted at several hospital sites in Minneapolis, MN and surrounding communities. Outcome assessment visits will be conducted at a hospital clinical outcomes center at the primary study site, in participant homes, by phone, or using secure video conferencing. MACE and mortality outcomes will be tracked via outcome assessments and medical records review.

### Participants

ACS is defined as a diagnosis of unstable angina, ST-elevation myocardial infarction, or non-ST-elevation myocardial infarction. ACS will be confirmed by coronary angiogram before enrollment. If diagnosis in the medical record was unclear or contradictory, a study cardiologist will review the case. Only Type I myocardial infarctions will be included (Type II myocardial infarctions caused by demand ischemia will be excluded). Myocardial infarction caused by spontaneous coronary artery dissection (SCAD) was included. We included SCAD cases because SCAD is associated with high rates of both smoking [39] and depression [40]. SCAD is also increasingly considered under the umbrella of ACS [41] and some have argued it should considered a cause of Type I myocardial infarctions [42]. Further, because SCAD occurs primally in women [41], including SCAD will improve gender balance in our study.

Individuals will be identified for recruitment while hospitalized on an inpatient unit for an ACS event. The hospitalization context can include both the initial emergency treatment for the ACS event, as well as subsequent hospitalizations within 30 days of the ACS event related to ACS treatment (e.g., returning to the hospital for bypass surgery). Additional inclusion criteria include (1) smoking ≥ 1 cigarette per day before hospitalization, (2) willing to consider quitting smoking at discharge, (3) age of 18–75, (4) English fluency, (5) lives within a 1.5 h drive from study site or willing to do all post-discharge study procedures remotely, (6) has regular access to a telephone or is willing and able to use a study issued cell phone, and (7) willing to consent to all study procedures. See Table 2 for a history of changes to inclusion criteria during the study.

**Table 2:** Major protocol changes

Description	Date
Remuneration for counseling sessions 3–6 was added to protocol based on participant feedback that their participation was limited because phone calls were using up limited cell phone minutes	11/12/2018
Provision of study issued cell phones to participants without other telephone access was added to the protocol to increase recruitment	7/31/2019
Protocol was updated to allow for recruitment of patients hospitalized for ACS related treatment or complications within 30 days of an index ACS event. This change was made to increase recruitment, particularly of those who are discharged quickly following index ACS but have a planned return for revascularization treatment	9/24/2019
Participants living more than a 1.5 h drive away were allowed to participate if they were willing to do all post-discharge study procedures remotely, including bio-verification of smoking status via mail. This change was made to increase recruitment and because a device (i.e., the Bedfont iCO Smokerlyzer®) that allows for remote measurement of exhaled carbon monoxide had recently become available	9/24/2019
The protocol was updated to allow recruitment and consent up to 1-week post-discharge. This change was made due to COVID-19 related hospital inpatient unit access restrictions	5/4/2020
Procedures allowing for remote (i.e., without in person contact) written consent were added to the protocol. These changes allowed for the consent discussion via phone with signed documents mailed, scanned, or dropped off/picked up with no in person contact. This change was made due to COVID-19 related restrictions	5/4/2020
The original protocol hypothesized group differences in intermediate cardiac outcomes (i.e., blood pressure, HDL-cholesterol, fibrinogen, and high sensitivity C-reactive protein). These measures were originally collected via in person blood draws at outcome assessments. Due to COVID-19 restrictions on in person visits, collection of these outcomes was suspended on 3/17/2020. Collection of these outcomes was dropped permanently from the study protocol in 7/8/21 due to significant missing data and continued participant preference for remote visits due to COVID-19	3/17/2020 (collection suspended)
	7/8/21 (removed from protocol)
The protocol was updated to allow for digital signing of consent and HIPAA forms allowable (via REDCap). This change was made to streamline the remote consent process	8/7/2020
Secure Zoom video conferencing allowed for all contacts as needed	8/7/2020

Exclusion criteria will be (1) limited mental competency (i.e., Mini Mental Status Exam [43] score ≤ 20); (2) a current exacerbation of serious mental illness that would interfere with study participation or current suicidality; (3) expected discharge to hospice or > 50% chance of 6 month mortality per the Global Registry of Acute Coronary Events [44] (GRACE) risk calculator; and (4) currently attending counseling targeting depression or smoking cessation. Note that we did not require patients to have symptoms of depression at baseline because a significant percentage of ACS patients with depression experience symptom onset post-ACS [11, 45, 46]. We enrolled those smoking ≥ 1 cigarette per day based on ample evidence that even one cigarette a day has deleterious effects on cardiac health [47].

### Pre-randomization procedures

#### Screening

Participants will be recruited from inpatient units at several Minneapolis area hospitals (Hennepin County Medical Center, MHealth Fairview University of Minnesota Medical Center, MHealth Fairview Southdale, Abbott Northwestern, North Memorial). We will assess most inclusion/exclusion criteria via review of the patient’s electronic medical record and self-report. If ACS diagnosis status is unclear in the medical record, staff will review the case with a study cardiologist at that site. If there is an indication of cognitive impairment in the patient chart or if the patient exhibits potentially impaired cognitive function at time of recruitment, staff will complete the Mini-Mental State Exam [43] (MMSE; scores of ≤ 20 will be excluded).

#### Informed consent

Participants that appear to qualify based on medical record review will be approached by research staff. At first contact, study staff will provide an overview of procedures and ask if the participant is willing to be screened. If the patient agrees to be screened and meets inclusion/exclusion criteria, they will be invited to participate. Informed consent will be obtained from participants before any further study procedures. As part of the consent process, we will ask five questions about the study to confirm patient understanding. Patients who answer fewer than four questions correctly (after two tries) will be considered to lack the capacity to consent. All participants will provide informed consent per protocols approved by the Hennepin Healthcare Research Institute IRB (HSR#17-4375). Any changes to the consent protocol will also be approved by the IRB before implementation. All participants will provide written informed consent either via paper or e-consent. It is expected most patients will be consented prior to hospital discharge using paper forms. However, if interested patients are discharged from the hospital before the consent process is complete (see Timing of Pre-Randomization Activities Relative to Hospitalization Section below), patients will be offered the option of documenting their informed consent electronically through Research Electronic Data Capture [48, 49] (REDCap). Only research study staff with training regarding the consent process will obtain consent. The full consent document is provided in additional file 2.

#### Baseline measures

After consent, participants will complete an initial baseline assessment (i.e., “Baseline 1”; See Table 1) consisting of interview questions and self-report questionnaires. Socio-demographic variables collected by self-report at baseline will be used to describe the sample and include: age, gender, ethnicity, employment, and income. Medical history will be assessed through self-report and chart review and will include: (1) diagnosis, treatment, and length of stay for current admission; (2) detailed history of cardiac disease, events, procedures, and treatments (e.g., revascularization); and (3) co-morbid conditions. Smoking history, including current and past smoking, age of smoking onset, and average number of cigarettes per day will be assessed via a self-report. Use of other tobacco products will be assessed by self-report. Nicotine Dependence will be measured using the Fagerström Test for Nicotine Dependence [50] (FTND). Motivation to quit will be measured using the contemplation ladder [51]. A second, brief baseline assessment will be conducted 1 week following hospital discharge and immediately prior to randomization to condition (i.e., “Baseline 2,” See Table 1). This assessment will include assessments of motivation to quit/stay quit [51] and nicotine withdrawal as baseline variables [52]. Both Baseline 1 and Baseline 2 assessments include assessments of outcome variables (see Table 1 for timing, see outcome measures sections below for a full description of all outcome variables).

#### Timing of pre-randomization activities relative to hospitalization

All participants will be initially identified as potentially qualifying during their in-patient stay for ACS or ACS treatment and, in general, participants will complete screening, consent, Baseline 1, and the initial treatment session before discharge from the hospital. However, discharge timing following an ACS can be unpredictable and there are several clinical activities that must occur before discharge (e.g., phase 1 cardiac rehabilitation); thus, the window for research activities is often narrow. Further, COVID-19 related restrictions (starting on 3/17/2020) on research staff entering inpatient rooms have varied across our recruitment sites and over time. Due to these challenges, when necessary, patients will be allowed to complete activities meant to be completed inpatient up to 1 week following discharge (see Table 2).

#### Randomization

Randomization will occur 1 week after hospital discharge, immediately after the Baseline 2. We will stratify randomization on elevated symptoms of depression (Patient Health Questionnaire [53] [PHQ-9] score of ≥ 10 vs. < 10 as measured immediately prior to randomization), use of the study provided nicotine patch between discharge and randomization (i.e., using patch vs. not using patch), and recruitment site (i.e., recruited at primary hospital site vs. hospitals subcontracted for recruitment). The randomization sequence will be generated using a stratified permuted block randomization procedure, with small, random sized blocks. The sequence was generated by the study biostatistician. Only the biostatistician and database manager will have access to the randomization sequence and all other study personnel will remain blind to this sequence. Counselors will randomize participants immediately before initiating Session 2 by entering the participant’s identification number, PHQ-9 score, use the nicotine patch, and recruitment site into REDCap [48, 49]. The REDCap interface will indicate the treatment assignment and the counselor will immediately inform the participant of their treatment condition.

### Treatment conditions

#### Behavioral activation treatment for cardiac smokers (BAT-CS)

BA has its underpinnings in the behavioral model of depression [54], which purports that depression results from reduced engagement in positively reinforcing activities. For participants, this condition will be referred to as the *“Smoking Cessation and Mood”* condition. All of BAT-CS will be delivered by tobacco treatment specialist counselors. BAT-CS consists of 1 in-hospital session and 5 post-discharge sessions over 12 weeks. Session 1 will always occur following informed consent, enrollment, and the Baseline 1 assessment. In general, Session 1 will occur during the ACS hospitalization. However, as noted above, enrollment will be allowed up to 1 week following discharge if not feasible during the inpatient stay. In these cases, Session 1 will occur as soon as possible following discharge and will be conducted in-person at the study outcomes center, in-person at the patient’s home, by video conference, or by telephone (based on participant preference, participant ability to travel, and relevant COVID-19 related restrictions at the time). The same delivery options will be offered for session 2 (1 week post discharge). Sessions 3–6 (at 3-, 6-, 9-, and 12-weeks post-discharge) will only be offered by phone or video conference.

If medically approved (i.e., cleared by participant’s provider or study cardiologist), 8 weeks of nicotine patches will be offered at no cost to the participant. Disinterest in or medical contraindication to the patch is not an exclusion criterion for the study. Patch dosing will follow clinical guidelines and FDA instructions. Patches will be provided to participants in the hospital with instructions to use study patches at discharge. Participants recruited after discharge will be provided with patches as soon as possible after discharge via home drop-off or overnight mailing. If a participant is not interested in nicotine patches at the time of enrollment, 8 weeks of patches will be provided upon request at any time during treatment (i.e., 12 weeks post-discharge) with medical clearance. Participants reporting significant side effects due to the patch will be advised to discontinue use until they can review with their medical provider, or a study cardiologist can provide guidance.

##### Session 1 (50 mins)

Session 1 will occur in hospital whenever possible. Session 1 will be guided by the current clinical practice guidelines [55] and the National Cancer Institute’s *Clearing the Air* patient workbook [56], which the participant will keep. The counselor will provide a strong and personalized recommendation to quit and will discuss (1) past attempts to quit, reframing these attempts as learning opportunities, (2) the risks of smoking and benefits of quitting in general and for cardiac patients, (3) personal reasons for quitting, (4) how to recognize and problem-solve smoking triggers, (5) how to elicit support for quitting, (6) tips for managing cravings, (7) planning for a quit date (i.e., how to remove cigarettes and paraphernalia), and (8) the safety and efficacy of the nicotine patch. Per treatment guidelines [55], brief motivational strategies will be provided to those who remain ambivalent about quitting. Participants ambivalent about quitting may set intermediate goals (e.g., reduction of cigarettes; stop smoking inside home).

##### Session 2 (50 mins)

Session 2 will begin assessment of smoking status, nicotine patch use, and degree of motivation to quit/stay quit, and a discussion of between session mood. If the participant is abstinent, the counselor will reinforce their effort, discuss times since last session when participant struggled with cravings, review strategies for managing triggers and cravings, and problem-solve upcoming challenges to staying quit. If the participant is smoking, the counselor will reinforce any efforts that the participant made (i.e., cutting down), discuss cause of relapse and problem-solve potential solutions to avoid that cause in the future, and utilize brief motivational strategies to prompt another quit attempt. If the participant is willing, the counselor will assist in planning a new quit attempt.

The counselor will also introduce BA mood management in session 2. The importance of addressing depressed mood after a hospitalization for ACS and the rationale for BAT-CS (i.e., loss of valued activities causes depressed mood; poor mood can interfere with smoking cessation) will be discussed with the participant. Next the counselor will conduct a values assessment to guide future activation goals [57]. Specifically, the counselor will review several life areas (e.g., family, work, etc.) and ask the participant to identify any strongly held values in each area (e.g., Family: “I value being a responsible grandparent”). The discrepancy of these strongly held values with continued smoking will be discussed (e.g., “How will continued smoking detract from grandparenting?”). The participant’s reported values will be returned to throughout treatment as motivators for adherence to activation goals and smoking cessation. The counselor will also assess for pleasant/valued activities the participant has given up or severely restricted post-ACS and discuss potential replacements for these losses.

The participant and counselor will then collaboratively set 2–4 activation goals to be completed before the next session. These goals will be guided by the values assessment, replacement of any restricted activities, and consistency with a non-smoking lifestyle. All goals will be within current physical abilities and will take into account that the participant is trying to quit smoking (e.g., goals should not involve increased contact with smoking triggers). The counselor will help problem-solve barriers to goal completion using established BA techniques (e.g., stimulus control, accessing social support) to maximize likelihood of completion. At the end of session 2 the counselor will provide a written list of stated values and immediate activation goals. With the participant’s permission, study staff will send an email or text message between sessions to remind and encourage the patient to complete their goals.

The participant will also be provided with an American Heart Association patient education booklet entitled *After Your Heart Attack*, focused on recommendations for life after an ACS [58]. This booklet is for the participant to review outside of session and is provided to the BAT-CS group so that all participants receive the same health education materials.

##### Sessions 3–6 (30 min)

Sessions 3–6 will follow the same basic format: (1) assess nicotine patch use, smoking status and degree of motivation to quit/stay quit, and provide brief motivational strategies for those still smoking or continued support to those quit; (2) assess and discuss mood since last session; (3) review adherence to activation goals from the previous session; (4) choose 2–4 activation goals that are pleasant and/or in line with the participant’s values and explicitly schedule these; (5) problem-solve barriers to completion of new goals, and (6) offer an automated email or text message reminder of goals. During the final counseling call (week 12), rather than assigning new activation goals, the counselor will guide the participant in continuing to work towards activation goals and smoking abstinence following termination of counseling.

#### Control condition

Consistent with the Pragmatic Model for Comparator Selection in Health-Related Behavioral Trials [59], we chose our control group based on the purpose of the study. Because the purpose of this trial is to demonstrate if providing BA based mood management specifically (rather than increased supportive counseling contact more generally) improves outcomes, we chose an attention control condition.

For participants, this condition will be referred to as the “*Smoking Cessation and Health*” condition. All control condition components will be delivered by the same tobacco treatment specialist counselors providing BAT-CS sessions. The control condition fully controls for contact time in post-discharge sessions by providing cessation counseling plus health and wellness education. All control condition sessions will parallel BAT-CS sessions in terms of session length, timing, and medium of administration.

Participants randomized to control will receive an in-hospital session 1 (50 min.) identical to that received in the BAT-CS condition, including being offered 8 weeks of the nicotine patch free of charge when medically appropriate (see detailed description of Session 1 content above). At each post-discharge session (1, 3-, 6-, 9-, and 12-weeks post-discharge) control participants will receive the same smoking cessation-focused content as the BAT-CS group, but will not be provided with any BA components (i.e., no values assessment, structured goal setting, or focus on the interaction of smoking and mood).

During session 2 in the control condition, participants will revive smoking cessation content, followed by health education focused on life after an ACS. This education will be guided by a booklet from the American Heart Association entitled *After Your Heart Attack* [58], which provides an overview of why a heart attack happens, what happens after a heart attack (i.e. medications, lifestyle changes, cardiac rehabilitation), emotional responses to having a heart attack, and provides strategies for preventing future cardiac events, including medication adherence, weight management, smoking cessation, limiting alcohol intake, and engaging in physical activity. For sessions 3–6 in the control condition, participants will choose one of the following health topics to discuss: (1) Heart Healthy Nutrition, (2) Fruits and Vegetables, (3) Sleeping Well, (4) Screenings and check-ups, (5) Staying Hydrated, and (6) Communication with your providers. Previous work by our team using similar health education content showed it has no significant effect on mood during smoking cessation [60]. Each education component will be explicitly linked to cardiac health as the content is introduced. To maintain the integrity of our independent variable, we chose topics that are relevant to cardiac health, but do not directly address mood or smoking cessation.

### Outcome assessments

Comprehensive outcomes assessments will be conducted in person, by phone, or using video conference at end of treatment (12 weeks after discharge) and 6, 9, and 12 months after discharge. Abbreviated phone assessments will be conducted by phone at 18-, 24-, 30-, and 36-months post discharge. Assessments through 12 months post-discharge will include biochemical verification of self-reported smoking status. Abbreviated assessments after 12 months will only collect self-reported smoking status.

#### Smoking outcomes

The primary outcome related to smoking will be 7-day point prevalence abstinence (i.e., no smoking at all in the past 7 days, not even a puff), which will first be assessed by self-report. If participants self-report ≥ 7-day abstinence, we will bio-verify their self-report. Saliva cotinine will be the primary bio-verification method (saliva cotinine of less than < 10 ng/ml will be considered abstinent) [61]. Saliva will be obtained from participants using the passive drool collection method and processed by Salimetrics using the salivary cotinine assay to determine the cotinine level present in the sample [62]. Participants will be instructed by research staff on how to prepare for and provide a sample of approximately 0.5 ml of passive drool. For those using nicotine replacement or noncombustible tobacco products, a carbon monoxide (CO) breath sample will be used to verify abstinence (CO < 6 ppm will indicate abstinence [61, 63]). Saliva samples and CO breath samples will be collected by mail if a face-to-face meeting is not possible. In person CO samples will be collected by research staff using the Covita Micro + Basic Smokerlyzer device [64] and remote CO samples will be collected using the Covita iCO Smokerlyzer [65], with telephone guidance provided by study staff. Secondary smoking outcomes will be 30-day point prevalence abstinence, continuous abstinence since discharge, and time from discharge to lapse (i.e., first puff) and relapse (i.e., smoking 7 consecutive days or in two consecutive 7-day periods). Participants who report using other combustible tobacco products will be considered smoking. Use of e-cigarettes and other non-combustible tobacco products (e.g., chewing tobacco) will be assessed at each timepoint and reported, but will not affect determination of abstinence.

#### Mood and quality of life outcomes

The primary outcome of depressed mood will be assessed using the PHQ-9 [53], a well-established self-report measure of depression in medical populations. A PHQ-9 score ≥ 10 is a well-established cut off indicating likely clinical depression. Secondary mood and quality of life outcomes will include the 10-item Center for Epidemiologic Studies Depression Scale [66] (CESD-10). The CESD-10 is included as a secondary outcome because it has a timeline of only 1 week and thus can give a better measurement of post-discharge, pre-randomization depressed mood (i.e., mood in the time period between Session 1 and randomization)**.** We will measure positive and negative affect using the 10-item Positive Affect Negative Affect Scales [67] (PANAS). Low levels of positive affect represent one aspect of anhedonia (i.e., low levels of positive emotions). We will also measure reactive anhedonia (i.e., blunted response to the occurrence of pleasurable events) using the Snaith Hamilton Pleasure Scale [68, 69] (SHAPS). Stress will be assessed using the Perceived Stress Scale [70] (PSS-4). Health Related Quality of Life will be measured using the 12-Item Short Form Health Survey [71] (SF-12).

#### Cardiac health outcomes

Exercise capacity will be assessed using the self-report Duke Activity Status Index [72] (DASI). Major Adverse Cardiac Events [73–75] (MACE; i.e., hospitalization for unstable angina, urgent coronary revascularization, and myocardial infarction according to standard American Heart Association criteria) and all-cause mortality will be tracked for up to 3 years (36 months post-discharge)*.* We will obtain records from any hospitalization or outpatient revascularization reported during assessments or follow-up phone calls. For any participant lost to follow-up, we will review medical records at recruitment hospital sites and mortality databases, as well as contact participant provided emergency contacts to obtain MACE/mortality status. Independent coding of MACE events will be completed by two study cardiologists using a structured coding form. They will meet to resolve any coding disagreements and a third cardiologist will resolve any disagreements on which a consensus cannot be reached. See Table 2 for details of changes to cardiac health outcome measures due to COVID-19.

Adverse events and patient safety will be tracked throughout the study, including hospitalizations, CO readings indicating toxic environmental exposure, unexpected side effects to the nicotine patch, and suicidality. All staff will be trained in a safety protocol which includes responses to both physical (e.g., CO readings indicating high environmental exposure) and psychological safety events (suicidality). A safety committee consisting of site PIs and an independent investigator otherwise not associated with the study will meet yearly to discuss all adverse events and other safety issues. A DSMB was not required by the funder at the time of study initiation.

#### Hypothesized BA mediators

Engagement in pleasant and valued activities will be measured using the 9-item Behavioral Activation for Depression Scale [76] (BADS) and the frequency items on the 20-item Pleasant Events Schedule [77, 78] (PES).

#### Treatment acceptability

Will be assessed quantitatively using the Client Satisfaction Questionnaire [79] (CSQ-8).

#### Potential confounders

We will collect data at all follow-ups on potential confounders of treatment effects so we can control for these variables as needed in analyses (i.e., data on dose of treatment received, cardiac rehabilitation attendance, depression or smoking counseling or medication use outside of the study, engagement in exercise, diet, and adherence to medications).

#### Blinding

Only the study statistician and data manager will have access to the allocation to treatment order prior to randomization. Outcomes assessors will be blind to treatment condition throughout the study. They will conduct end-of-treatment (12 week) and 6-, 9-, and 12-month assessments, and bi-annual calls monitoring for MACE/mortality. If the assessor learns a participant’s randomization assignment through any means (i.e., becomes unblinded), another assessor will take over that participant’s remaining assessments/calls. The study cardiologists will be blind to treatment condition throughout the study and will code records for the occurrence and type of MACE.

#### Retention and incentives for participation

Based on our pilot work and previous clinical trials in which we have obtained follow up completion rates of 85–90% (e.g., R01AT006948; K23HL107391; R44DA022167) [80, 81], we expect to complete follow-up assessments with at least 80–85% of participants. A comprehensive maintenance program similar to our previous studies will be used to ensure this level of retention.

Participants will receive $50 at each primary assessment completed [Baseline 1, Baseline 2, End-of-Treatment (12 weeks), and 6-, 9-, and 12-month follow-ups]. Transportation costs (taxi service, bus fare, mileage, and/or parking vouchers) to an in-person assessment will be paid for by the study. Participants will be paid $5 each for completion of counseling sessions 3, 4, 5, and 6 and a $20 bonus if that participant completes all four of these sessions. Remuneration for counseling sessions 3–6 was added (see Table 2) during the trial based on participant feedback that phone calls were using up limited cell phone minutes. For each brief phone assessment completed from 18 to 36 months post-discharge, participants will be compensated $10.

We will provide participants with study marketing materials (e.g., coffee mugs, water bottles, tote bags) that will include the study name, logo, phone number, and email. We will offer one item at Baseline 2 (1 week post-discharge) and one item at any follow-up assessment visit (12w, 6 m, 9 m, 12 m) attended in person. New Years and mid-Summer cards with the study logo will be sent to all study participants through the duration of their participation. Incentives and provision of marketing materials will be identical in each condition. We will send text, email, and/or mail reminders in advance of each assessment. We will obtain three “emergency contacts” (i.e., who will know how to reach the participant should their phone number be out of service) from each participant and these contacts will be updated as needed throughout the study.

Participants who do not have regular access to a phone or those who lose access to a phone during the first year of enrollment will be offered a study issued cellphone to complete study activities. Upon completion of the 12-month outcomes assessment, participants using study issued cellphones will be compensated $20 for returning the phone to study staff.

#### Interventionist background and training

Counseling will be completed by individuals with a Bachelor’s or Master’s degree in health education or a related field. All counselors will complete tobacco treatment specialist training, read materials on tobacco treatment [55], BA [54], and ACS [82–84], listen to mock sessions completed by clinical psychologists, and complete mock sessions with other study staffs. Use of tobacco treatment specialists greatly improves cost effectiveness, real world applicability, and potential for future dissemination.

#### Intervention fidelity

We will implement intervention fidelity procedures developed by the NIH Behavioral Change Consortium [85]. We will use detailed written manuals for each intervention condition to minimize any potential for contamination between conditions. Counselors will be trained in each manual by two clinical psychologists.

Counselors will audio record all sessions in both conditions and complete a self-report treatment fidelity checklist following each session in both conditions. Supervisors will review audio recordings of all sessions of each counselor’s first patient in each condition and a random 10% of all subsequent sessions in both conditions. Supervisors will complete the treatment fidelity checklist for each session they review. Counselors will have weekly supervision with clinical psychologists during which any barriers to fidelity or drift from the manual will be addressed.

#### Data management

Data will be managed by a PhD biostatistician and a data manager. All staff on the study will be trained in proper data management and security. Paper files will be stored in a locked cabinet within a locked room. All digital data will be stored on either secure, password protected institutional servers or in REDCap. Participant tracking will be conducted using custom software on a SQL server. Randomization will be completed in REDCap. REDCap will also be used to record informed consent and HIPAA authorization when completed electronically.

#### Power

The proposed study is fully powered for both Aim I primary outcomes (i.e., biochemically confirmed 7-day point prevalence abstinence and depression symptoms on the PHQ-9 over 12 months post discharge). Based on existing data at the time of study design [36, 38, 86], we expect 7-day point prevalence rates to differ by 8% (29% in control, 37% in BAT-CS) at 12 months, representing an odds ratio of 1.4 at 12 months. We will analyze our data longitudinally (see below), thus we base our sample size on the longitudinal effect across the 4 follow-ups (i.e., end-of-treatment and, 6, 9, and 12 months post discharge). We expect a *longitudinal* odds ratio of 1.33 ending with an 8% between-group difference at 12 months as described above. It is important to note that experts have proposed that increasing rates of smoking cessation in ACS patients by even 5% would have a large impact on public health [87]. Given the assumptions above and an alpha level of 0.05, a total sample size of 324 participants would be required in order to have sufficient (80%) power to test the *longitudinal* effect on intent-to-treat 7-day point prevalence abstinence over 1 year post-discharge. This sample will be more than sufficient (power > 80%) to detect even small longitudinal effects on depression symptoms over 1 year (i.e., with 324 participants we have 80% power to detect between group effect of f squared = 0.03).

#### Primary outcome analyses

We will estimate the effect of the interventions on smoking cessation (biochemically verified 7-day point prevalence abstinence) at end-of-treatment and follow-ups (6, 9 and 12 months) using a longitudinal regression model implemented with generalized estimating equations (GEE) with robust standard errors [91, 92]. Specifically, we will regress smoking status on intervention group (BAT-CS vs. control) and potential covariates (cardiac rehabilitation attendance, cessation treatment outside of the study, other baseline variables will be included as covariates if they are both not balanced by randomization and are correlated with the outcome under consideration at *p* < 0.10 level) using binomial errors, a logit link function, and a working unstructured correlation to accommodate within-subject correlation.

We will use a series of longitudinal mixed effects regression models to estimate the effect of BAT-CS versus control on depressed mood (PHQ-9) at end-of-treatment and subsequent follow-ups, controlling for baseline depression, cardiac rehabilitation attendance, depression treatment outside of the study, and any other variables not balanced by randomization that are correlated with depressed mood at follow-ups at a modest *p* < 0.10 level. Models will include a subject-specific intercept to adjust for repeated measurements within participant over time. Modeling is done using a likelihood-based approach and thus makes use of all available data (on the intent to treat sample) without directly imputing missing outcomes, to produce consistent estimates of the regression parameters.

For primary outcomes (i.e., biochemically verified 7-day point prevalence abstinence and PHQ-9) we will conduct a per-protocol analysis (with parallel models to those described above) to assess differences between groups among those who completed a substantial number of treatment sessions. Per protocol will be defined completing at least four treatment sessions in both treatment groups.

#### Secondary outcome analyses

A similar modeling strategy to that described above for 7-day point prevalence abstinence will be used to assess between group differences in 30-day point prevalence abstinence and continuous abstinence. We will estimate the effect of interventions on time to first lapse (i.e., first puff) and first relapse (i.e., smoking 7 consecutive days or in two consecutive 7-day periods) using survival analysis [93]. Each participant contributes two outcome variables to the model: T*_i_, time to first lapse/relapse and C_i_, censoring time (T*_i_ < C_i_ for those who lapse/relapse before the end-of-observation and T*_i_ > C_i_ for those who don’t lapse/relapse before end-of-observation or discontinue the study protocol before lapsing/relapsing). The response is considered to be min {T*_i_, C_i_}. Using a Cox model [94] , we can write the hazard function (which can be thought of as the number of lapses/relapses per participant-day of follow-up time) as a function of a baseline hazard rate λ_0_(t) and covariates X(t), including intervention assignment.

Parallel longitudinal mixed effects regression models to those used to evaluate between group differences in PHQ-9 will be used to evaluate changes CESD-10, PANAS, SHAPS, PSS, SF-12, and DASI.

We will test the effect of BAT-CS versus control on the time to occurrence of MACE and all-cause mortality over the 36-month follow-up (see Fig. 2). Time to event data will be modeled using a similar survival analysis method to that described above, but importantly, will also control for duration of follow-up as this will differ by participant and will likely be associated with the outcome. We will conduct separate survival analyses for MACE and all-cause mortality but will also analyze a composite of both to maximize power.

#### Mediator analyses

We will explore potential mediators of intervention effects on depressed mood and smoking cessation. We will consider two mediation models: Model 1 will consider engagement in pleasant and valued activities (i.e., BADS, PES) as mediators of the intervention effect on depressed mood; Model 2 will consider depressed mood and engagement in pleasant/valued activities (i.e., BADS, PES) as potential mediators of the intervention effect on smoking cessation. For Model 1, we will use a multiple mediation approach, in which all potential mediators are tested simultaneously using a product of coefficients method [95] with bootstrapped standard errors (5000 samples with replacement). We will estimate the path coefficients (*a* path: effects of intervention on changes in mediators over time and *b* path: effects of changes in the mediators on depression over time, controlling for baseline), as well as the indirect effect of intervention (*ab* path: effect of intervention on depression through the mediators). Interest is in estimating the path coefficients, effect sizes, and confidence intervals, rather than strict hypothesis testing. In Model 2, we will use a similar approach to estimate the path coefficients and indirect effects of intervention on smoking cessation over time through changes in depressed mood and engagement in pleasant/valued activities over time.

#### Interaction of depressed mood and smoking over time analyses

Finally, we will explore the temporal interaction of smoking and multiple aspects of mood over 12 months post-ACS using an autoregressive, cross-lagged regression model, in which trajectories of mood, smoking status and their cross-lagged effects are studied simultaneously over time and effects of BAT-CS versus control on these relationships can be estimated.

##### Missing data

All analyses (other than the per protocol analyses detailed above) will be on the intent-to-treat sample (everyone randomized will be included in the final analysis) under various assumptions about the missing data mechanism. Sensitivity to these assumptions will be tested. Specifically, we will gather follow-up information and reasons for dropout regardless of protocol completion. We will compare the robustness of our findings using two statistical approaches for handling missing data. First, we will use inverse probability weighting with propensity scores. This is a two-step method: (1) using logistic regression, the probability of missingness is modeled as a function of baseline covariates and baseline values of the outcome and (2) the inverse of the propensity scores (predicted probabilities of dropout from the first step) serve as weights in our regression model of the outcomes. Provided the data is missing at random (MAR) or that the probability of missingness can be fully explained by observable data, this approach produces asymptotically unbiased estimates. To allow for the possibility that the MAR assumption may not hold, we will also use a second approach, pattern mixture models, in which the distribution of the outcome is assumed to follow a mixture of two distributions: one for those who complete follow up and another for those who do not.

## Discussion

Approximately 400,000 ACS patients who smoke are discharged from the hospital annually in the US [1–3]. Thus, even modest improvements in the rate of smoking cessation post-ACS could save thousands of lives [87]. Moreover, results of this study may also provide insight into the role of smoking in the relationship between depression and cardiac morbidity and mortality in ACS patients and provide a model for how to investigate the role of other health behaviors in this relationship (e.g., diet, physical activity, medication adherence). The work of integrating treatments that can address both psychological distress and health behavior changes into existing medical systems and discovering sustainable models is crucial to long-term intervention impact. If this study shows BAT-CS to be efficacious for smoking, we plan to propose a multi-site effectiveness study focused on integration into ongoing hospital systems, cost-effectiveness, and long-term sustainability.

## Supplementary Information

**Additional file 1. SPIRIT guidelines checklist.**

**Additional file 2. Informed consent document.**

## Data Availability

The deidentified dataset will be made available 18 months after study closure to interested investigators who submit a written request. We will publish the results in academic journals and results will be posted on clinicaltrials.gov.

## Abbreviations

ACS: Acute coronary syndrome
BA: Behavioral activation
BADS: Behavioral activation for depression scale
BAT-CS: Behavioral activation for cardiac smokers
CO: Carbon monoxide
CSQ-8: Client satisfaction questionnaire
DASI: Duke Activity Status Index
FTND: Fagerström test for nicotine dependence
GEE: Generalized estimating equations
GRACE: Global registry of acute coronary events
MACE: Major adverse cardiac events
MAR: Missing at random
MMSE: Mini-mental state exam
PANAS: Positive affect negative affect scales
PES: Pleasant events schedule
PHQ-9: Patient health questionnaire
PSS-4: Perceived stress scale
RCT: Randomized control trial
SCAD: Spontaneous coronary artery dissection
SF-12: Short form health survey
SHAPS: Snaith hamilton pleasure scale
