# Supplementary material for: Integrated smoking cessation and mood management following acute coronary syndrome: Protocol for the post-acute cardiac event smoking (PACES) trial
Source: Addict Sci Clin Pract. 2023 May 12;18:29. doi: 10.1186/s13722-023-00388-9 (PMC10175930; doi:10.1186/s13722-023-00388-9)
Supplement: Supplementary file 1 — Additional file 1. SPIRIT guidelines checklist. [file 13722_2023_388_MOESM1_ESM.docx]

Appendix A: Consent form

**Consent to Participate in a Research Study**

TITLE OF STUDY: Post Acute Cardiac Event Smoking (PACES) Study
The purpose of this paper is to give you basic information about a research study. As you read these pages, feel free to ask questions. Being a part of this study is your choice, so please think about the information in this paper carefully. If you choose to be a part of the study, you can sign a consent, or agreement, at the end of these pages.

**1. INVESTIGATOR(s) CONDUCTING THIS STUDY**

***Who will be in charge of this study?***

Andrew Busch, Ph.D., Principal Investigator, Hennepin Healthcare, Department of Medicine, 701 Park Ave., S9-309, Minneapolis, MN 55415 (612)873-6681

Woubeshet Ayenew, MD., Co- Investigator, Hennepin Healthcare, Department of Medicine, 701 Park Ave., Minneapolis, MN 55415, (612)873-2875

Michael Miedema, MD., Co-Investigator, Abbott Northwestern Hospital and Minneapolis Heart Institute Foundation, 920 E. 28th Street, Suite 260, Minneapolis, MN 55407, (612) 863-3900

Sandra Japuntich, Ph.D., Co-Investigator, Hennepin Healthcare, Department of Medicine, 701 Park Ave., S9-303, Minneapolis, MN 55415
(612)873-6856

Beth Bock, Ph.D., Co-Investigator, The Miriam Hospital, 164 Summit Avenue, Providence RI, 02906, (401)793-8020

Shira Dunsiger, Ph.D., Co-Investigator/Biostatistician, Brown University School of Public Health, Department of Behavioral and Social Sciences, Box G-S121-8, Providence RI, 02912, (401)863-6550

Wen-Chih Wu, MD, Co-Investigator/Cardiologist, The Miriam Hospital, 164 Summit Avenue, Providence RI, 02906, (401)273-7100

Susan Everson-Rose, Ph.D. Co-Investigator, University of Minnesota, 717 Delaware St SE, Suite 166, Minneapolis, MN 55414, (612)624-0468

Prabhjot S. Nijjar, MD, Co-Investigator/Cardiologist, University of Minnesota, 420 Delaware St. SE, MMC 508, Minneapolis, MN 55455, (612)626-1391

Brett Carlson, MD, Co-Investigator, North Memorial Health Hospital, 3300 Oakdale Avenue North, Robinsdale, MN 55422, (763) 581-5400

**2. SOURCE OF SUPPORT
*Who is funding this research study?***

The sponsor, the National Heart, Lung, and Blood Institute (NHLBI) is paying for the costs of this study.

**3. SITE OF THE RESEARCH STUDY**

***Where will this study be done?***

This research study will be done at Abbott Northwestern Hospital (part of the Allina Health system), University of Minnesota/Fairview Hospital, North Memorial Health Hospital, Hennepin County Medical Center (Hennepin Healthcare; part of the Hennepin Health System), and Hennepin Healthcare’s research arm, the Hennepin Healthcare Research Institute (HHRI). Participants will be enrolled during inpatient hospital stays at Abbott Northwestern Hospital, University of Minnesota/Fairview Hospital, North Memorial Health Hospital, and Hennepin Healthcare. In person contacts with study staff after you leave the hospital will occur at the Berman Center, a clinical research center within the HHRI located in the Parkside Professional Building at 825 S 8th St, Minneapolis, MN 55404. If you are physically unable to travel, study contacts may be conducted in your home or your residential facility, such as a rehabilitation center or nursing home, or other preferred location that you specify. Some contact with study staff will be over the phone.

Study staff and investigators at The Miriam Hospital and Brown University, both in Providence, RI, will be involved in data management and analysis.

**4. PURPOSE OF THIS RESEARCH STUDY
*Why is this research study being done?***

You are being asked to take part in a research project because you are a cigarette smoker and you recently experienced a cardiac event. This study is researching two approaches to helping those who had had a cardiac event quit smoking. You will be randomly assigned to participate in one of these treatments. Randomization means that you are put into a group by chance. It is like flipping a coin. Which group you are put in is being done by a computer. Neither you nor the researcher will choose what group you will be in. You will have an equal chance of being in each group. We expect to enroll 324 subjects into this study.

The treatment phase of this study will occur over the next 12 weeks. Primary assessments will occur over the next year. Brief phone assessments will begin in 18 months from now and continue for up to 5 years from now. We may access your medical records up to 6 years from now.

**5. ELIGIBILITY**

***Who is being asked to be part of this research study?***

You are being asked to be part of this study because you are between the ages of 18 and 75, have had a cardiac event that is considered an acute coronary syndrome, and smoke at least 1 tobacco cigarette per day.

**6. PROCEDURES**

***What procedures will be done for this research study?***

Treatment: If you take part in this study, you will receive about 1 hour of counseling in the hospital to help you quit smoking. This counseling session may happen over the phone or by Zoom, at the Berman Center, in your home, or other preferred location that you specify if you are discharged early. Following this session, you will be randomly assigned to receive 5 sessions of either **a)** smoking cessation and mood management counseling or **b)** smoking cessation and health counseling. Randomization will occur 1 week after you are discharged from the hospital. All participants will have access to a free 8-week supply of the nicotine patch if their medical provider approves.

If you are assigned to the smoking cessation and mood condition, you will receive 5 counseling sessions over 12 weeks after you leave the hospital. The first will be about one hour and will be conducted in person, by phone, or by Zoom. The next 4 sessions will occur over the phone or by Zoom and will take about 30 minutes. Sessions will be focused on helping you to quit smoking and manage your mood and stress. You will collaboratively agree on between session goals with your counselor.

If you are assigned to the smoking cessation and health condition, you will receive 5 counseling sessions over 12 weeks after you leave the hospital. The first will be about one hour and will be conducted in person. The next 4 sessions will occur over the phone or Zoom and will take about 30 minutes. Each session will begin with a discussion of your smoking and nicotine patch use. The rest of sessions will then focus on a health topic.

All counseling sessions will be audio-taped for quality control and training purposes. These tapes will be kept strictly confidential. Hennepin Healthcare staff will review these tapes for quality control purposes.

Primary Assessments over next 12 months: As part of the study, you will complete a 30-45 minute baseline assessment (“baseline assessment 1”) when you are still in the hospital. This assessment may happen over the phone or Zoom, at the Berman Center, in your home, or other preferred location that you specify if you are discharged early. Baseline assessment 1 will consist of self report questions about your background, your smoking, your mood, and your health. All further primary assessments will occur at the Berman Center located in the Parkside Professional Building at 825 S 8th St or other preferred location that you specify. This visit may be conducted by phone or Zoom if required.

Next, you will complete a second 20-30 minute baseline assessment 1 week after hospital discharge (“baseline assessment 2”) at the Berman Center or by other preferred method that you specify (in-person visit, phone, or Zoom). During baseline assessment 2 you will be asked to provide saliva and breath samples and will also complete questionnaires. Some baseline data may also be pulled from your medical record at the hospital where you received treatment for your cardiac event.

Immediately following treatment (about 12 weeks after hospital discharge), and at 6, 9, and 12 months following discharge, you will complete a 60 minute assessment at the Berman Center or by other preferred method that you specify (in-person visit, phone, or Zoom). During these assessments you will be asked to provide saliva and breath samples and will also complete questionnaires. You will also be asked about any recent hospitalizations and we will obtain and review records from any reported hospitalization.

Saliva and breath samples are collected at baseline assessment 2 (1 week after discharge) and all primary follow-up assessments (12 weeks and 6, 9, and 12 months after discharge). You will provide a saliva sample of about .5 teaspoon total. Some of this sample will be used to test for levels of cotinine (a nicotine metabolite). Neither you nor your provider will get feedback on the results of this test. Remaining saliva may be frozen for future analysis. If you report not smoking at any assessment you will provide a breath sample (by exhaling into an air monitor). This will be used to test for the level of carbon monoxide in your breath.

If using Zoom for any study visits these visits will be conducted over the web and will use wifi or data, any costs incurred will not be paid for by the PACES study.

Brief Phone Assessments: Starting 18 months following discharge from the hospital after this cardiac event, we will call you to complete a brief phone call regarding your health, hospitalizations, smoking, and mood. We plan to make these calls at 18, 24, 30, 36, 48, and 60 months post discharge. Our current funding allows us to complete these calls through 36 months for early participants and through 18 months for final participants. Thus, additional calls will be dependent on obtaining additional funding.

Hospitalization/record review. During all assessments, if you report that you were hospitalized for any cardiac event or had a cardiac catheterization procedure, we will obtain those records for review and coding. If you were treated at a Hennepin Healthcare, UMN/Fairview Hospital, North Memorial Health Hospital or Allina affiliated hospital or clinic, study staff will pull your records from our medical record system. If you were treated at an outside clinic or hospital we will obtain those records for review.

Emergency Contacts. If we are unable to reach you repeatedly for any study contact, we will call the emergency contacts you provide to ask about how to reach you and about your health and mortality status. If we are unable to contact you, we will also review public death records and Hennepin Health, UMN/Fairview Hospital, North Memorial Health Hospital, and Allina hospital system records to see if you have passed way or if you have been hospitalized or received cardiac catheterization at an affiliated hospital or clinic. We will ask your emergency contacts if they know if and where you have been hospitalized and if you have passed away. If your emergency contacts report you have been hospitalized or received cardiac catheterization procedure since your last contact with the study, we will request those records for review.

**7. RISKS, DISCOMFORTS, AND INCONVENIENCES**

***What are the possible risks, side effects, discomforts, or inconveniences of this research study?***

The risks of this study are similar to those present for any person attempting to quit smoking.

The discomforts inherent to quitting smoking include withdrawal symptoms such as mood swings, anxiety, irritability, decreased concentration, restlessness, excessive hunger, and trouble sleeping. These symptoms are not dangerous and usually only last about one to two weeks after quitting.

There may be some risk to using the nicotine patch among those who have recently experienced a cardiac event. National guidelines state that the nicotine patch should be “used with caution” among some groups of cardiac patients, including those who have had a heart attack in the past 2 weeks. Thus, if you want to use the nicotine patch as part of this study, we will first ask your provider if the benefits of using the nicotine patch outweigh any risk. If you do receive the patch from us and your medical status changes, you are responsible for checking with a provider about whether or not it is still medically safe for you to continue to use it. If you use the patch, you may have a) some skin irritation or itching where you put the patch on your body, b) dizziness, rapid heartbeat, or upset stomach if the dose is too large or if you smoke while using the patch, and c) vivid or strange dreams. You could have an allergic skin reaction to the patch, but this is not as likely to happen. Chest pain is a rare side effect of the patch. If this occurs, you should immediately stop using the patch and contact your healthcare provider. If you are pregnant or become pregnant during the study, there may be risks to you and your unborn child if you use the patch. You cannot use the patch if you are pregnant, trying to get pregnant, or breast feeding a child. If you find that you are pregnant, you should stop using the patch and inform your provider immediately. It is safest not to smoke when using the nicotine patch.

You may feel uncomfortable about answering assessment questions and talking with your counselor as part of the study. You may feel uncomfortable providing saliva or breath samples. You will be asked about some potentially sensitive issues including your mood, smoking history, and your medical history as part of this study.

**8. REPRODUCTIVE AND PREGNANCY ISSUES
*What is important to know about being a part of this study and pregnancy?***

A pregnant woman could potentially participate in this study if other criteria are met. However, a pregnant participant would not be offered the nicotine patch. If a participant became pregnant during the study, she could continue to participate, but would be asked to stop using the nicotine patch.

**9. HEALTH BENEFITS
*What are the possible health benefits to you or to others from your being part of this research study?***

You may or may not benefit from participating in this study. You may successfully quit smoking or cut down on the number of cigarettes you smoke due to study counseling, which may improve your health. Likewise, your mood may improve due to your participation in the study.

**10. ALTERNATIVE TREATMENTS**

***What treatments or procedures are there for you if you decide not to be part of this research study?***

Alternative forms of smoking cessation and mood treatment may be available to you, including prescription medication and counseling that are not included in this study. Both the American Cancer Society and the American Lung Association offer free and/or low cost programs to help people stop smoking. You can also speak with your medical providers about other options.

**11. CONFIDENTIALITY
*Who will know that you are part of this research study?***

Any information that could be used to identify you will be treated in strict confidence to the extent allowed by law. Nevertheless, some uses and disclosures of your information are necessary to conduct the study. If you agree to be part of this study, you will also be allowing the uses and disclosures of your private health information as needed for the purposes of this study as described in this consent.

“Private health information” means information that identifies you and is collected:

- during this study;
- from your past and current medical records maintained by your regular health care providers (including, if applicable, Hennepin Healthcare, UMN/Fairview Hospital, North Memorial Health Hospital, and/or Abbott Northwestern Hospital), to the extent the information is relevant to this study or to your eligibility for this study; or
- from any payment records relating to items or services furnished to you during this study.

By signing this consent, you are agreeing that your private health information may be disclosed to and used by:

- the doctors and other health care providers involved in this study;
- their staff;
- the research centers involved (HHRI; Minneapolis Heart Institute Foundation; University of Minnesota/Fairview Hospital; North Memorial Health Hospital; The Miriam Hospital);
- members of the Hennepin Healthcare, UMN/Fairview Hospital, North Memorial Hospital Health, and Allina Human Subjects Research Committees/Institutional Review Boards;
- the sponsor of this study and its agents; and
- monitors from the United States Government and/or Food and Drug Administration (FDA).
- If you are hospitalized or receive a cardiac catheterization procedure at an outside hospital or clinic, we will request these records. This request will disclose to these outside hospitals and clinics that you are participating in this study.

The findings of this study may be used for scientific meetings, written reports, and publications, but no information that could be used to identify you will be disclosed for these purposes.

Once your private health information has been disclosed to a third party, federal privacy laws may no longer protect it from re-disclosure. However, anyone obtaining access to your private health information under this consent must agree to protect your information as required by this consent.

This consent to use your private health information as described above does not expire. However, if you later change your mind, you can revoke this consent by writing to Dr. Andrew Busch saying that you no longer wish to allow your private health information to be used for this study. If you revoke your consent, you may no longer be able to participate in the study. Moreover, we cannot undo uses or disclosures of your private health information that have already taken place in reliance on your prior consent.

You will not be allowed to see your study data while the study is in progress. However, after the study is finished you may see this information upon written request.

**12. COSTS ASSOCIATED WITH THE RESEARCH STUDY
*Will your insurance provider or you be billed for any costs of any treatments, medicines, or procedures done as part of this research study?***

Neither you nor your insurance provider will be billed for the costs of any of the medicines, procedures, or treatments used just for this research study (i.e., screening, assessments, counseling treatment) explained earlier. You will be billed in the regular way for any medicine/procedures/treatments done as part of your routine medical care. If you believe you have received a bill in error during the research study, contact the project manager, Michelle Chrastek at (612)873-5364.

**13. COMPENSATION AND MEDICAL TREATMENT FOR ANY STUDY-RELATED INJURY
*If you are injured from being part of this research study, what should you do and who will pay for it?***

Not applicable. This research involves no more than minimal risk

**14. COMPENSATION FOR PARTICIPATION
*Will you be paid for being part of this research study?***

You will receive $50 for each primary assessment you fully complete. These occur at time of recruitment, 1 week, 12 weeks, and 6, 9, and 12 months after discharge. For the telephone treatment sessions: Session 3, Session 4, Session 5, and Session 6 you will be compensated $5 for each call and will receive a $20 bonus for completing ALL telephone treatment sessions. Compensation for these sessions will be distributed at your 12 week primary assessment visit. So if you complete all 5 primary assessment visits and the 4 telephone treatments sessions you will receive $340. We will reimburse your parking and public transit costs for attending assessments. Cab fare will be reimbursed if you are not physically able to drive/use transit. You will receive $10 for each fully completed brief phone assessment that takes place for calls beginning at 18 months.

**15. NEW FINDINGS
*Will you be told of any new information or new risks that may be found while this study is going on?***

In every research study, there may be risks we do not expect. You will be told about any important new information that may cause you to change your mind about being part of this study.

**16. FREEDOM TO PARTICIPATE AND WITHDRAW
*Is being part of this research study voluntary? Can you decide to stop being in this research study at any time?***

Being part of this research study is your choice. You do not have to be part of this study. You can agree to be in the study now and change your mind later. Your decision to stop being in the study will not affect your regular care. Your doctor's attitude toward you will not change.

If you decide to stop being in the study, the principal investigator or his staff may discuss with you a more limited participation in this study such as still collecting information from your medical records after you stop your direct participation. If you agree at that time, to such continued limited participation, that agreement will be noted in your records.

**17. PROCEDURES FOR ORDERLY WITHDRAWAL OR REMOVAL FROM THE STUDY
*What would happen if you decide to stop being part of this study or if you are removed from this study?***

You may be taken out of the study if the researchers decide that you staying in the study would be unsafe for you or others in any way, or if the study is canceled. If you decide to stop being part of the study or if you are removed from the study for any reason, you will stop completing study activities immediately.

**18. CONTACT INFORMATION FOR QUESTIONS
*Who should you contact if you have questions?***

A description of this clinical trial will be available on http://www.ClinicalTrials.gov, as required by U.S. law. This website will not include information that can identify you. At most, the website will include a summary of the results. You can search this website at any time.

If you have any problems, concerns, or questions about the study or your rights as a subject in this research study, want to obtain information, or want to offer input, and want to talk to someone other than the study doctor, you can call the Office of Human Subjects Research at Hennepin Healthcare Research Institute at (612) 873-6881.

If you have any questions before signing this consent, please be sure to ask them now. During the study, if you have any questions, concerns, or complaints for the study doctor, please call Dr. Busch at (612) 873-6681.

**VOLUNTARY CONSENT FORM**

I have either read the attached consent or it has been read to me.

By signing this form, I do not give up any of my legal rights or release anyone involved in this research study from their responsibility for negligence.

By signing this form, I agree to be part of this research study and consent to the use of my private health information as described in Section 11 (“Confidentiality”) of the attached consent.

A signed copy of this consent will be given to me.

Subject's / Legally Authorized Representative's Printed Name and Signature

Date

I certify that a copy of this form has been provided to the above-named subject.

Explained by Printed Name, Title, and Signature

_________________________

Date
